# Cognitive-perceptual deficits and symptom correlates in first-episode schizophrenia

**DOI:** 10.4102/sajpsychiatry.v23i0.1049

**Published:** 2017-08-31

**Authors:** Riaan M. Olivier, Sanja Kilian, Bonginkosi Chiliza, Laila Asmal, Petrus P. Oosthuizen, Robin Emsley, Martin Kidd

**Affiliations:** 1 Department of Psychiatry, Faculty of Medicine and Health Sciences, Stellenbosch University, South Africa; 2 Department of Statistics and Actuarial Sciences, Stellenbosch University, South Africa

## Abstract

**Background:**

Thought disorder and visual-perceptual deficits have been well documented, but their relationships with clinical symptoms and cognitive function remain unclear. Cognitive-perceptual deficits may underscore clinical symptoms in schizophrenia patients.

**Aim:**

This study aimed to explore how thought disorder and form perception are related with clinical symptoms and cognitive dysfunction in first-episode schizophrenia.

**Setting:**

Forty-two patients with a first-episode of schizophrenia, schizophreniform or schizoaffective disorder were recruited from community clinics and state hospitals in the Cape Town area.

**Methods:**

Patients were assessed at baseline with the Rorschach Perceptual Thinking Index (PTI), the Positive and Negative Syndrome Scale (PANSS) and the MATRICS Cognitive Consensus Battery (MCCB). Spearman correlational analyses were conducted to investigate relationships between PTI scores, PANSS factor analysis-derived domain scores and MCCB composite and subscale scores. Multiple regression models explored these relationships further.

**Results:**

Unexpectedly, poor form perception (X- %) was inversely correlated with the severity of PANSS positive symptoms (*r* = -0.42, *p* = 0.02). Good form perception (XA%) correlated significantly with speed of processing (*r* = 0.59, *p* < 0.01), working memory (*r* = 0.48, *p* < 0.01) and visual learning (*r* = 0.55, *p* < 0.01). PTI measures of thought disorder did not correlate significantly with PANSS symptom scores or cognitive performance.

**Conclusions:**

Form perception is associated with positive symptoms and impairment in executive function during acute psychosis. These findings suggest that there may be clinical value in including sensory-perceptual processing tasks in cognitive remediation and social cognitive training programmes for schizophrenia patients.

## Introduction

Thought disorder and visual-perceptual deficits in schizophrenia have been well described in the literature,^1^ but their relationships with other psychopathology and cognitive functions remain unclear. These relationships are important as they may help us to better understand the symptom expression of the illness, as well as its underlying pathophysiology.

Numerous studies have described visual-perceptual deficits in schizophrenia involving abnormalities in visual scanning and eye movements, visual form recognition, figure-ground perception, and visual backward masking and impaired motion perception.^2^ However, little is known about the relationship between visual-perceptual deficits and clinical symptoms or cognitive deficits.^2^ Although visual-perceptual deficits have been observed to be significantly correlated with negative symptoms, research findings have been inconsistent.^3^

Cognitive deficits are well described in schizophrenia and most studies have shown that they have modest correlations with negative and disorganisation symptoms in schizophrenia and weak correlations with positive symptoms.^4,5^ The cognitive mechanisms underlying thought disorder remain unclear. Some authors have argued that the supportive cognitive functions of memory, attention and executive function are considered to play a contributory role in the development of thought disorder.^6^

A significant advance in cognitive assessment has been the incorporation of the MATRICS (Measurement and Treatment Research to Improve Cognition in Schizophrenia) Cognitive Consensus Battery (MCCB) which was endorsed by the National Institute of Mental Health (NIMH) as the standard assessment of cognition in all schizophrenia clinical trials.^7^ Although less well-studied, the Rorschach Perceptual Thinking Index (PTI) has been proposed as a sensitive measure of thought disorder and perceptual accuracy.^8^ The PTI offers the advantage of tapping into the subtleties of thought disorder and offers a quantifiable perceptual accuracy index, which provides a measureable unit of the degree of thought disorder and form perception impairment.

In this study, we sought to examine thought disorder and form perception with the PTI in a cohort of acutely psychotic patients with a first-episode of schizophrenia. We further explored the relationships between thought disorder and form perception with other components of psychopathology and cognitive function. Assessments were conducted when subjects were untreated or minimally medicated. We hypothesised that the severity of thought disorder and poor form perception would be significantly correlated with disorganisation and negative symptom domains, as well as with poor cognitive function.

## Research methods and design

### Subject selection and clinical assessment

Subjects were recruited over a period of 18 months from first admissions to Tygerberg and Stikland hospitals, and community clinics in Cape Town, South Africa. All subjects were between the ages 16 and 45 and had received previous treatment with an antipsychotic drug of 4 weeks or less. Subjects were excluded if they had (1) received previous treatment with depot antipsychotic drugs, (2) evidence of a significant physical illness, neurological condition or less than 7 years formal schooling, (3) current and ongoing substance abuse at screening, (4) a psychiatric disorder other than schizophrenia, schizophreniform or schizoaffective disorder according to the Diagnostic and Statistical Manual of Mental Disorders (DSM-IV-TR) or (5) were not fluent in English or Afrikaans.

Written informed consent was obtained from all subjects after explaining the purpose, procedures, risks and benefits of the study. Informed consent was obtained from the legal guardians of those subjects who did not have the capacity to give consent.

Baseline clinical assessments included a Structured Clinical Interview (patient version) for the DSM-IV-TR and the Positive and Negative Syndrome Scale (PANSS). Investigators underwent training for PANSS rating and inter-rater reliability was ≥ 0.7. The PANSS forced five-factor model was applied to arrive at negative, positive, disorganised (or cognitive), excited and anxiety or depression factors.^9^

### Cognitive assessment

Cognitive performance was assessed within the first week of starting treatment. The MCCB consists of 10 individual subtests making up seven domain scores: (1) Speed of processing, (2) Attention or vigilance, (3) Working memory, (4) Verbal learning (5) Visual learning, (6) Reasoning- and problem-solving and (7) Social cognition. The MCCB Computer Scoring Program was used to convert raw scores into T-scores for the seven cognitive domains, and to compute an overall composite T-score. Data entry on the computer was done with age-and-education corrected norms for the US standardisation population.^7^ Two investigators (R.M.O. and S.K.) were registered psychologists and received training in the MCCB test administration and scoring. The other authors were responsible for subject screening, clinical ratings and assistance with data analysis.

### Assessment of thought disorder and form perception

We chose the PTI which is an index score derived from the Rorschach Comprehensive System (RCS) for the measurement of thought disorder and form perception.^8^ The PTI measures two-dimensional constructs, that is, thought disorder and form perception, and comprises five-criterion-rated scores: (1) Good form perception (XA% < 0.70) and good form perception to whole and large detail (WDA% < 0.75), (2) Poor form perception (X- % > 0.29), (3) Deviant verbalisations and responses reflecting Illogical Thinking (Sum2 > 2) and responses reflecting Fabulised Thinking and Implausible Combinations (FAB2 > 0), (4) Low number of responses (*R* < 17) and sum of six weighted scores reflecting Unusual Thinking and Illogical Combinations (WSum6 > 12) or (*R* > 16) and (WSum6 > 16) and (5) Poor human form perception (M- > 1) or poor form perception (X- % > 0.40). The PTI Total score has a range from 0 to 5 with a suggested cut-off of 3 or more, indicating the likelihood of psychosis.^8^ We calculated *Lambda* (ratio of pure form responses to the frequency of all responses) which reflects a tendency to defensiveness or masking of psychopathology. We excluded all score sheets with *R* less than 14 because of questionable validity in these protocols.^10^ We did not exclude protocols with *Lambda* higher than 0.99 because of the high number of participants whose *Lambda* values exceeded 0.99. An independent rater coded the RCS variables and entered the scores into the Rorschach Interpretation Assistance Program (RIAP5) to arrive at the PTI total and subcategory scores. Inter-rater reliability of 13 critical PTI variables was tested by a second-blinded rater who coded and scored 20 randomly selected Rorschach protocols. Intraclass correlation reliability analysis was conducted with intraclass correlation coefficients (ICC) ranging from 0.55 (WSum6), 0.74 (F-) and 0.80 (F+). Considering Cicchetti’s (1994) guidelines, these coefficients are acceptable and fall in the range of fair, good and excellent, respectively.^11^

### Treatment

Subjects were assessed at baseline or within the first week of initiation of antipsychotic treatment. For those who were considered too ill to be accurately assessed, testing was postponed until they were more settled. No anticholinergic or sedative medication was given 12 h before cognitive assessments.

### Statistical analysis

Statistical analyses were performed with Statistica version 12 (Statsoft) software. A 5% significance level (*p* < 0.05) was used as the guideline for determining significant differences. Spearman correlational analyses were conducted to investigate relationships between PTI total and subcategory scores, PANSS factor-derived scores and MCCB composite and domain scores. Multiple linear regression analyses were conducted with the PTI indices of thought disorder (WSum6) and form perception (XA%) as dependent variables, and as predictor variables we included the PANSS factor scores, PANSS total and MCCB composite score. We included age, gender, education and lifetime drug use as covariates in our analyses to control for their possible moderating effects.

## Ethical considerations

The authors obtained approval from the Human Research Ethics Committee of Stellenbosch University’s Faculty of Medicine and Health Sciences to conduct this study.

## Results

The sample included 42 individuals (8 females and 34 males) with a mean (s.d.) age of 24 (6.9) and 10.7 (2.1) years of education and duration of untreated psychosis of 237 (269) days. DSM-IV-TR diagnoses were schizophreniform disorder (*n* = 22, 53%), schizophrenia (*n* = 19, 45%) and schizoaffective disorder (*n* = 1, 2%). Most patients were of mixed ethnicity (*n* = 28, 67%). There were also 11 (26%) black South Africans and 3 (7%) white South Africans.

Table 1 presents the mean (s.d.) scores for the PTI total and sub scores, MCCB composite and subscale scores and PANSS total and factor-derived domain scores for the sample. Patients were moderately ill (*PANSS* total mean = 89.1, s.d. = 16.9) with cognitive performance falling between 2 and 3 SD’s below the norm (*T-score mean* = 50, s.d. = 10). The PTI total (2.2) was raised, above the suggested norms, with impairments in the perceptual accuracy indices (XA% < 70, X- % > 29). Indices of thought disorder (WSum6, Sum2 and FAB2) were raised, but did not reach the suggested threshold for psychosis (threshold for PTI criteria [d] = *R* > 16 and WSum6 > 16). *Lambda* was raised (*Lambda* > 0.99).

**TABLE 1:** MATRICS Cognitive Consensus Battery, Perceptual Thinking Index and Positive and Negative Syndrome Scale scores for the 42 patients.

Variable	Mean	s.d.
PTI Total score	2.2	1.5
Lambda	2.3	3.2
WSum6 %, (raw score)	59.8 (14.1)	47.6
FAB2 % (raw score)	0.48 (0.14)	1.6
Sum2 % (raw score)	6.2 (1.5)	7.9
M- % (raw score)	2.5 (0.6)	4.2
XA %	60.0	18.3
WDA %	62.6	18.6
X- %	38.3	17.8
PANSS Total	89.1	16.9
PANSS P	23.1	6.9
PANSS N	22.3	7.7
PANSS D	21.4	7.5
PANSS E	7.4	3.9
PANSS A/D	11.2	5.8
MCCB composite, T-score	15.5	15.8
Attention or vigilance	26.3	11.4
Speed of processing	22.7	14.2
Verbal learning	36.2	7.4
Visual learning	32.5	15.4
Working memory	25.8	15.2
Reasoning- or problem-solving	33.6	8.9
Social cognition	26.8	15.1

MCCB, MATRICS Cognitive Consensus Battery; PTI, Perceptual Thinking Index; Lambda, ratio of pure form responses compared with all responses; WSum6, sum of 6 weighted scores for Unusual Thinking and Illogical Combinations; FAB2, Fabulised Thinking and Implausible Combinations; Sum2, Deviant Verbalisations and Illogical Thinking; M-, Poor Human Form perception; XA, Good Form perception; WDA, Good form perception to whole and large detail; X-, Poor form perception; PANSS, Positive and Negative Syndrome Scale; P, positive factor; N, negative factor; D, disorganisation factor; E, excited factor; A/D, anxiety or depression factor.

Spearman correlations between PTI measures and the MCCB subtest and composite scores are shown in Table 2. Poorer performance on good form perception was significantly correlated with more severe cognitive impairments in executive function (processing speed and working memory) and visual memory (XA) (*r* = 0.48–0.59, *p* < 0.01).

**TABLE 2:** Correlations between Perceptual Thinking Index measures and the MATRICS Cognitive Consensus Battery subtest and composite scores.

Variable	Attention vigilance	Speed of processing	Verbal learning	Visual learning	Working memory	Reasoning problem solve	Social cognition	MCCB composite
WSum6	−0.24	0.01	−0.06	−0.30	−0.16	0.06	0.12	−0.06
FAB2	−0.09	−0.04	0.02	−0.17	−0.08	0.12	−0.14	−0.11
Sum2	−0.12	0.02	0.08	−0.26	−0.13	0.03	0.12	0.01
M-	−0.03	−0.19	−0.22	−0.30	−0.20	0.17	−0.17	−0.13
XA	0.26	0.59**	0.34	0.55**	0.48**	0.15	0.33	0.58**
WDA	0.31	0.55**	0.37	0.53**	0.46*	0.18	0.33	0.55**
X-	−0.23	−0.57**	−0.32	−0.46*	−0.44*	−0.17	−0.35	−0.56**

MCCB, MATRICS Cognitive Consensus Battery;WSum6, sum of 6 weighted scores for Unusual Thinking and Illogical Combinations; FAB2, Fabulised Thinking and Implausible Combinations; Sum2, Deviant Verbalisations and Illogical Thinking; M-, poor human form perception; XA, good form perception; WDA, good form perception to whole and large detail; X-, poor form perception.

* *p* < 0.05;

** *p* < 0.01

Correlations between PANSS factor domain scores and the PTI measures and MCCB scores are provided in Table 3. More severe positive symptoms were significantly correlated with increased good form perception (XA) (*r* = 0.40, *p* < 0.05) and inversely correlated with decreased poor form perception (X-) (*r* = -0.42, *p* < 0.05). More severe negative and disorganisation symptoms were significantly correlated with decreased performance on cognitive tests of working memory (*r* = -0.42 and *r* = -0.47, *p* < 0.01), visual learning (*r* = -0.32 and *r* = -0.40, *p* < 0.05), and attention or vigilance (*r* = -0.39, *p* < 0.05 and *r* = -0.52, *p* < 0.01). Verbal learning was significantly correlated with disorganisation symptoms (*r* = -0.32, *p* < 0.05).

**TABLE 3:** Correlations between Perceptual Thinking Index and MATRICS Cognitive Consensus Battery scores and Positive and Negative Syndrome Scale factors.

Variable	Positive	Negative	Disorganisation	Excited	Anxiety or depression
WSum6	0.01	0.15	0.06	−0.09	−0.07
FAB2	0.01	0.22	0.21	−0.20	0.11
Sum2	−0.06	0.18	0.14	−0.06	−0.06
M-	−0.25	0.18	0.14	−0.06	−0.06
XA	0.40*	−0.10	−0.12	0.23	−0.06
WDA	0.42*	−0.16	−0.18	0.21	−0.10
X-	−0.42*	0.07	0.11	−0.20	0.08
A/V	−0.12	−0.39*	−0.52**	−0.05	0.05
SoP	−0.04	−0.25	−0.32	0.09	−0.18
VerbLrng	−0.06	−0.28	−0.33*	−0.12	−0.31
VisLrng	−0.19	−0.32*	−0.40*	0.21	−0.17
WM	0.03	−0.42**	−0.47**	−0.05	−0.02
RPS	−0.12	−0.12	−0.23	−0.32	−0.10
SC	−0.04	−0.01	−0.26	0.06	−0.22

A/V, Attention or vigilance; SoP, Speed of processing; VerbLrng, Verbal learning; VisLrng, Visual learning; WM, Working memory; RPS, Reasoning- and problem-solving; SC, Social cognition; WSum6, sum of six weighted scores for Unusual Thinking and Illogical Combinations; FAB2, Fabulised Thinking and Implausible Combinations; Sum2, Deviant Verbalisations and Illogical Thinking; M-, Poor human form perception; XA, Good form perception; WDA, Good form perception to whole and large detail; X-, Poor form perception.

* *p* < 0.05;

** *p* < 0.01

For the linear regression models, we calculated the predictive power of PANSS factor and MCCB composite scores on XA% (form perception) and WSum6 (thought disorder). PANSS positive and disorganisation symptoms were found to have a significant predictive ability (*R*^2^ = 0.318) on XA% (*b* = 0.653, *p* = 0.001) and (*b* = -0.627, *p* = 0.02), respectively.

The cognitive composite score had a significant predictive ability (*R*^2^ = 0.273, *b* = 0.582, *p* = 0.001) on XA%. We found no significant predictive ability of symptoms or cognitive performance on the Rorschach indices of thought disorder (WSum6).

## Discussion

In this study, we investigated thought disorder and form perception as assessed by the PTI and explored their associations with other psychopathological symptoms and cognitive function. By studying treatment-naive and minimally treated patients with first-episode schizophrenia, we minimised the potential confounding effects of disease chronicity and treatment. We found a substantial degree of thought disorder and visual-perceptual disturbances, although the mean PTI total of 2.2 (1.5) was slightly below the suggested psychosis threshold of three or more.^8^ However, our PTI scores are similar to those of a recent study investigating the PTI in an older sample of more broadly defined psychotic patients (mean age = 35.9[11.6], mean PTI total = 2.6[1.5]).^3^

### Perceptual Thinking Index and Positive and Negative Syndrome Scale scores

We did not find a significant correlation between PTI measures of thought disorder and other psychopathological symptoms. This study was conducted on a very small sample (*N* = 42), therefore the negative findings could be because of a Type II error. The study by Biagiarelli et al. on the other hand reported a significant relationship between thought disorder as assessed by the PTI and PANSS negative and positive subscales.^3^ While this study was conducted in a larger sample (*N* = 98), only 34 were assessed during the acute episode and 64 were ‘chronically treated and stable’. The Biagiarelli et al. study, which differed from ours in that it included a broader spectrum of psychotic disorders, was not a first-episode cohort and assessments were not conducted prior to treatment.^3^ Thus, we were unable to replicate the findings of Biagiarelli et al.^3^

Our finding of a significant correlation between good form perception and positive symptoms (*r* = 0.40, *p* < 0.05) and an inverse correlation between poor form perception and positive symptoms (*r* = -0.42, *p* < 0.05) was not anticipated. This association was confirmed by the finding that positive symptoms were significant independent predictors of XA% in the linear regression model (*R*^2^ = 0.318, *b* = 0.653, *p* = 0.001). This finding would appear at first sight to be contradictory, since accurate form perception on the Rorschach is also viewed as a measure of reality testing.^12^ Consequently, the question arises as to why subjects who achieved higher scores on measures of good form perception also scored higher on PANSS positive items that relate to delusions and hallucinations. Firstly, it is possible that individual items in the PANSS factor domain were responsible for the finding. However, the internal consistency of the PANSS positive items (P1, G9, P3, P6, P5, P6 and G12) was acceptable with a Cronbach’s standardised alpha coefficient of 0.73. Secondly, previous studies indicate that a high *Lambda* (ratio of pure form responses compared with all responses) with a threshold greater than 0.99 may mask the severity of thought and perceptual disturbances in psychosis.^8^ We found a raised *Lambda* of 2.3 which is significantly higher than the threshold. Our patients were acutely ill and quite impaired neuropsychologically which may make it difficult for them to engage effectively with the complexity of the Rorschach task. The possibility needs to be kept in mind that severity of illness impeded the ability of the participants to provide accurate responses. Finally, it could be speculated that our finding of better form perception in patients with more prominent positive symptoms reflects some kind of compensatory mechanism. It may be that cognitive processes react to more severe psychosis by heightening the overall perceptual capacity, either directly or indirectly by, for example, heightened awareness and attention span.

### Perceptual Thinking Index and MATRICS Cognitive Consensus Battery scores

We did not find support for an association between thought disorder and executive or working memory deficits, as has been previously reported.^13^ However, our finding that form perception was significantly correlated with processing speed (*r* = 0.59, *p* < 0.01), working memory (*r* = 0.48, *p* < 0.01) and visual memory (*r* = 0.55, *p* < 0.01), and that the MCCB composite score significantly predicted form perception (*R*^2^ = 0.273, *p* = 0.001), suggest an association between deficits in visual-perceptual processing and cognitive performance, in particular executive function and visual memory. While our study used a visual stimulus (Rorschach pictures) as a paradigm to measure form perception, future studies would do well to investigate multi-sensory modalities in FEP and their relation to cognition.

Our results confirm the findings of previous studies, reporting an association between PANSS negative and disorganisation symptoms with measures of cognitive performance.^4^ The association was evident across the cognitive domains of attention or vigilance, working memory, visual learning and verbal learning. This finding is similar to previous studies^5^ using the MCCB with the exception of reasoning- and problem-solving, for which we found no significant correlation. The strongest correlations emerged with the disorganisation factor (A/V) (*r* = -0.52, *p* < 0.01) and (WM) *(r* = -0.47, *p* < 0.01) which supports the work of Good et al.^14^ who suggests the renaming of this factor as the cognitive factor. The results of this study are consistent with other studies indicating significant cognitive impairment in FEP of a generalised nature. The very low MCCB composite scores of 2–3 standard deviations below the U.S. community age or gender corrected norm^7^ achieved in our study may reflect the illness severity at the time of testing or alternatively cross-cultural variations in MCCB test performance. Regarding the latter, to date the MCCB has not been standardised on a South African population. However, as already mentioned, in another study we conducted in the same population a control group of healthy volunteers was included. Results of that study confirmed that patients performed significantly worse than controls.^15^

Strengths of the study are that subjects were medication naive or minimally medicated and experiencing their first psychotic episode, thereby providing an opportunity to capture the salient cognitive, perceptual and thinking disturbances during acute psychosis. Limitations of this study include the small sample size, lack of a control group, language differences between raters and subjects and within the study sample. Lastly, the lack of South African norms for the cognitive tests and statistical findings which appear clinically counter-intuitive suggest that these results should be considered preliminary.

## Conclusion

Our findings suggest that the Rorschach PTI may not be a suitable instrument for assessing thought disorder, at least in acutely psychotic patients. However, it may be a novel approach in identifying disorders of thought perception and their relationships to both psychopathology and cognitive function. The associations that we found between form perception and cognitive dysfunction underscore the interconnectedness between visual-perceptual sensory input and higher cognitive processing, and in particular with prefrontal executive processing. This may have implications for treatment of schizophrenia. Cognitive remediation and social cognitive training could potentially be enhanced by including less demanding tasks involving auditory and visual sensory modalities. The inverse association between poor form perception and positive symptoms is intriguing and warrants further investigation. Clearly, future studies are needed to replicate these findings with larger samples and including healthy controls.
